# Homologous Recombination Deficiency Associated With Response to Poly (ADP-ribose) Polymerase Inhibitors in Ovarian Cancer Patients: The First Real-World Evidence From China

**DOI:** 10.3389/fonc.2021.746571

**Published:** 2022-01-06

**Authors:** Jing Ni, Wenwen Guo, Qian Zhao, Xianzhong Cheng, Xia Xu, Rui Zhou, Hongyuan Gu, Chen Chen, Xiaoxiang Chen

**Affiliations:** ^1^ Department of Gynecologic Oncology, The Affiliated Cancer Hospital of Nanjing Medical University, Jiangsu Cancer Hospital, Jiangsu Institute of Cancer Research, Nanjing, China; ^2^ Department of Pathology, The Second Affiliated Hospital of Nanjing Medical University, Nanjing, China; ^3^ Department of Chemotherapy, Jiangsu Cancer Hospital, Jiangsu Institute of Cancer Research, The Affiliated Cancer Hospital of Nanjing Medical University, Nanjing, China

**Keywords:** HRD, PARPi, predictive biomarker, ovarian cancer, Chinese population

## Abstract

Homologous recombination deficiency (HRD) is an approved predictive biomarker for Poly (ADP-ribose) polymerase inhibitors (PARPi) in ovarian cancer. However, the proportion of positive HRD in the real world and the relationship between HRD status and PARPi in Chinese ovarian cancer patients remain unknown. A total of 67 ovarian cancer patients who underwent PARPi, either olaparib or niraparib, were enrolled and passed inclusion criteria from August 2018 to January 2021 in the Affiliated Cancer Hospital of Nanjing Medical University. HRD status correlation with Progression-free survival (PFS) was analyzed and summarized with a log-rank test. Univariate and multiple cox-regression analyses were conducted to investigate all correlated clinical factors. Approximately 68.7% (46/67) patients were HRD positive and the rest 31.3% (21/67) were HRD negative. The PFS among HRD-positive patients was significantly longer than those HRD-negative patients (medium PFS 9.4 m vs 4.1 m, hazard ratio [HR]: 0.52, 95% CI: [0.38–0.71], p <0.001). Univariate cox-regression found that HRD status, Eastern Cooperative Oncology Group (ECOG) status, BRCA status, previous treatment lines, secondary cytoreductive surgery and R0 resection were significantly associated with PFS after PARPi treatment. After multiple regression correction, HRD status and ECOG were the independent factors to predict PFS (HR: 0.67, 95% CI: [0.49–0.92], p = 0.01; HR: 2.20, 95% CI: [1.14–4.23], p = 0.02, respectively). In platinum sensitivity evaluable subgroup (N = 49), HRD status and platinum sensitivity status remain significant to predict PFS after multiple regression correction (HR: 0.71, 95% CI: [0.51–0.98], p = 0.04; HR: 0.49, 95% CI: [0.24–1.0], p = 0.05, respectively). This is the first real-world study of HRD status in ovarian cancer patients in China, and we demonstrate that HRD is an independent predictive biomarker for PARP inhibitors treatment in Chinese ovarian cancer patients.

## Introduction

Ovarian cancer is the most lethal gynecological malignancy (1). Approximately 70% of the patients with ovarian cancer are diagnosed at an advanced stage. Most ovarian cancers patients are sensitive to platinum-based chemotherapy (2). How to prolong the platinum-free interval (PFI) is an important issue in ovarian cancer treatment. PARPi has changed the treatment pattern of ovarian cancer. Many clinical trials and real-world studies have confirmed that PARPi can significantly prolong the PFI of patients with ovarian cancer (3–6).

Poly (ADP-ribose) polymerase (PARP) plays an important role in DNA repair, maintenance of genome integrity, and regulation of various metabolic and signal transduction processes. PARP1 and PARP2 enzymes are activated after DNA damage that manifests mainly as single-strand breaks (SSBs), double-strand breaks (DSBs) or replication fork stalling (7); they also recognize and bind to the DNA fracture site and mediate DNA single-strand damage repair in tumor cells. In HRD-positive tumor cells, such as the BRCA mutation (BRCAmt) or other germline mutations in homologous recombination repair (HRR) pathway genes (e.g., RAD51 and ATM), single-strand DNA damage cannot be repaired, forming a synthetic lethal effect (8). Therefore, BRCAmt or HRD-positive tumor cells are more sensitive to PARPi (9). Previous studies also demonstrated that HRD-positive ovarian cancer patients had more significant clinical benefits from PARPi treatment (4, 6, 10).

At present, the FDA approved two commercial kits for HRD detection: FoundationFocus^®^CDx BRCA LOH (integrated to FoundationOne^®^CDx, shortened as F1CDx) and Myriad myChoice^®^CDx. The determination of HRD status is based on genomic scars. Cells with HRD had DNA repair dysfunction, which will lead to genome damage and leave genomic scars. FoundationFocus^®^CDx mainly evaluates HRD by loss of heterozygosity (LOH) with 16% as the threshold value that has been used for companion diagnostic (CDx) of rucaparib (11), while Myriad myChoice^®^CDx includes LOH, telomere allele imbalance (TAI), and large fragment migration (LST) with 42 as the threshold value that has been widely applied for CDx of niraparib and olaparib (4, 6, 10, 12). Approximately 50% of patients with high-grade serous ovarian cancer are HRD positive in the western population (13, 14).

In China, although four PARPi, namely, Olaparib, Niraparib, Pamiparib, and Fluzoparib, have been approved in ovarian cancer, HRD status has not been approved as a CDx biomarker, and there is no National Medical Products Administration (NMPA)-approved kit for HRD detection yet. Thus, the correlation of HRD status with PARPi therapeutic outcomes could be retrospectively studied in the real world. Our study aimed to perform HRD testing of ovarian cancer in the real world in China and correlate HRD status and clinical characteristics with therapeutic outcomes.

## Materials and Methods

### Study Population

**Figure 1:**
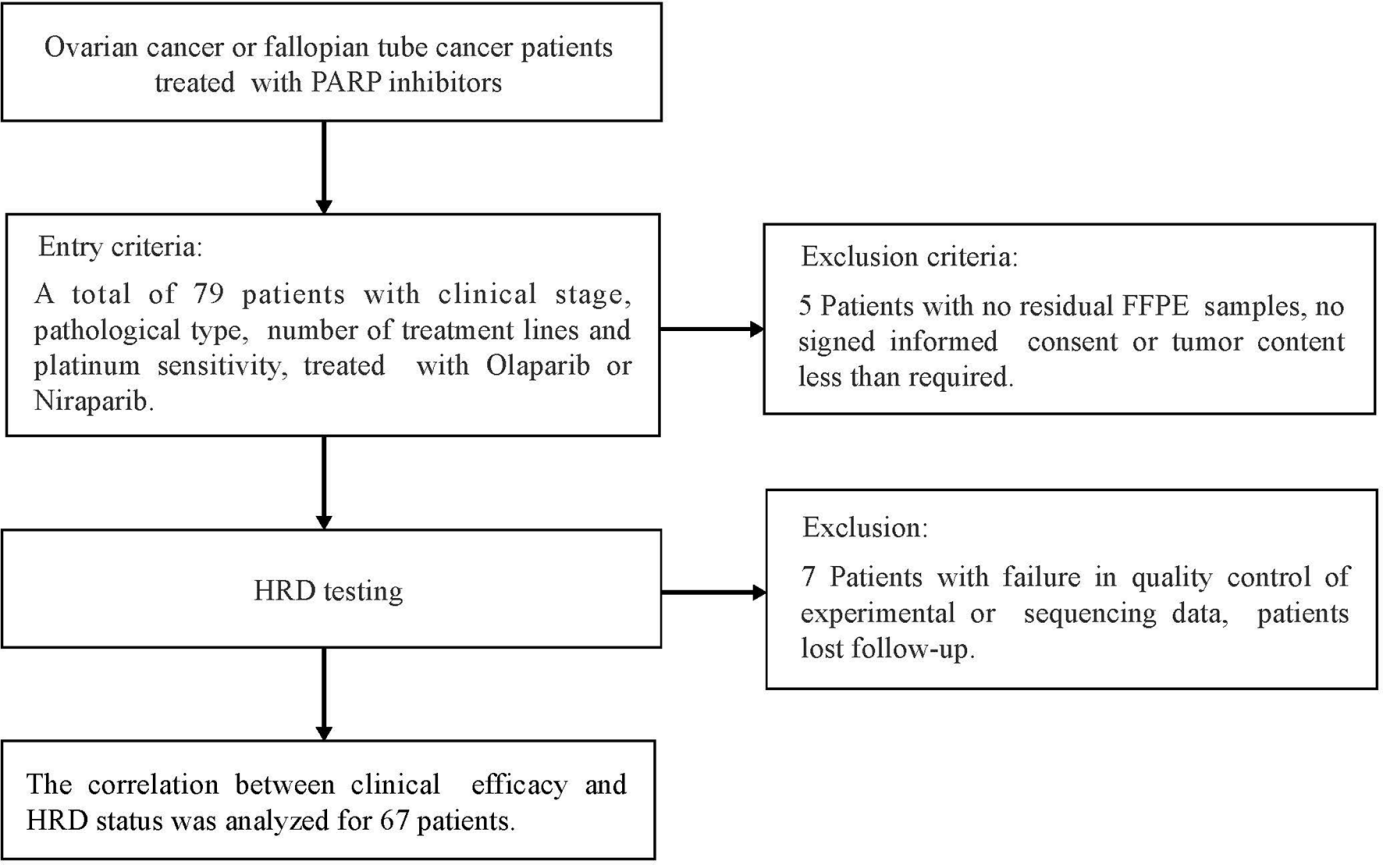
Flow Diagram for the Real world PARPi treatment data cohort, patient inclusion and exclusion criteria of patients used for validation.

Between August 2018 and January 2021, a total of 79 ovarian cancer or fallopian tube cancer patients were treated with PAPRi for more than four weeks, including olaparib and niraparib in the Affiliated Cancer Hospital of Nanjing Medical University, were recruited (NCT:05044091). If patients experienced serious adverse events (Grades 3–4), the dose reduction and interruption would be done according to drug instruction of olaparib or niraparib. Treatment continued until the occurrence of radiological progression, as assessed by the Response Evaluation Criteria in Solid Tumors 1.1(RECIST 1.1), unacceptable adverse events or death. Five patients with no qualified FFPE samples or no signed informed consent forms were excluded. Seven patients with failure of quality control of experimental or sequencing data or lost follow-up clinic information were further removed for data analysis. All methods were performed in accordance with the relevant guidelines and regulations by the ethics committee of the Affiliated Cancer Hospital of Nanjing Medical University. The study flowchart is shown in **Figure 1**.

### HRD Testing

The formalin fixation and paraffin embedding (FFPE) samples from cytoreductive surgery were obtained with the patients’ informed consent. DNA was extracted from FFPE biopsy/surgical specimens by MagPure FFPE DNA LQ kit (Kit# D6323). A total of 100 ng DNA (minimum 50 ng if the total DNA is less than 100 ng) was applied for library construction and 500 ng libraries were used for hybrid capture with an AmoyDx^®^ HRD panel, which selected coding sequences (CDS) regions for 54 genes and 72,000 single nucleotide polymorphisms (SNPs). The list of 54 genes is shown in **Supplementary Table 1**. The selected libraries were pooled and sequenced on the Illumina Novaseq6000 with >500× unique coverage for 54 genes and >100× for SNP loci.

Sequence data were processed using a customized analysis pipeline designed to accurately detect multiple classes of genomic alterations: base substitutions, short insertions/deletions with detection sensitivity at variant allele frequency (VAF) ≥5%. Detected mutations for the 54 genes were annotated according to the American College of Medical Genetics (ACMG) guideline and classified as pathogenic, likely pathogenic, variants of unknown significance (VUS), likely benign and benign (15).

BRCA mutation positive (BRCAmt) was defined as BRCA1 and or BRCA2 pathogenic or likely pathogenic mutation was detected in patients and otherwise is BRCA wildtype (BRCAwt). HRR mutation positive was defined as pathogenic and likely pathogenic mutations in the following 15 HRR pathway genes as BRCA1, BRCA2, ATM, BARD1, BRIP1, CDK12, CHEK1, CHEK2, FANCL, PALB2, PPP2R2A, RAD51B, RAD51C, RAD51D, RAD54L, HDAC2, and FANCA. HRD score was calculated by the sum of three types of genomically unstable events, namely, LOH, TAI, and LST as defined by (16–19). HRD score cut-off was validated by BRCAness status and maintained the same as Myriad myChoice^®^CDx cut-off 42 (19). HRD-positive was defined by BRCA mutation positive and/or HRD score ≥42.

### Clinical Assessments

All enrolled subjects received PARP inhibitor treatment, namely, olaparib or niraparib. Basic characteristics and follow-up information after PARPi treatment were collected. Serum CA125 and imaging examinations *via* computed tomography were performed on each patient at baseline, followed by a monthly examination of CA125 and bimonthly imaging examinations.

Adverse events (AEs) were graded according to the Common Terminology Criteria Adverse Events (CTCAE) V 5.0 to modulate the dosage. Progression-free survival was defined as the interval from the date of PARPi treatment to the date of disease progression or death from any cause, whichever occurred first.

### Statistical Analysis

The Fisher exact test was used to test the difference of categorical variables, namely, BRCA mutation status and HRR mutation status, and the odds ratios (ORs) and 95% confidence intervals (CIs) were also calculated. For progression-free survival (PFS) analysis, Kaplan–Meier curves were compared by using a log-rank test, and the hazard ratio (HR) was determined through a Cox proportional hazards regression model to test the correlations between different variables and PFS. Baseline variables that achieved a level of significance of P <0.2 in the univariable analysis were entered into multivariable models. All reported P-values were 2-tailed, and P <0.05 was considered to be statistically significant. Statistical analyses were performed with python lifelines package version 0.22.3.

## Results

### Patient Characteristics

In total, 65 ovarian cancer patients and 2 fallopian tube cancer patients were enrolled. The median age of patients was 55 years (range 31–80 years), and most of them were high-grade serous carcinoma (86.6%). In total, 49 patients were diagnosed with FIGO III stage (73.1%) and thirteen patients had FIGO IV stage (19.4%). Twenty-nine patients were R0 resected with no macroscopic disease (43.3%). Thirty-seven patients were Eastern Cooperative Oncology Group performance status (ECOG PS) 0 (55.2%). Twenty-three patients underwent neoadjuvant chemotherapy (NACT) (34.3%). Approximately 26.9% of patients underwent secondary cytoreductive surgery. A total of 27 patients have done ≤2 lines of therapy before PARPi therapy (40.3%). Platinum sensitivity status was evaluable in 49 (73.1%) of the patients. In total, 47 patients (70.1%) were treated with olaparib, and the remaining patients (29.9%) were treated with niraparib. The median follow-up time was 7.9 months. Patients’ clinic and pathological characteristics are summarized in **Table 1**.

**Table 1:** Baseline characteristics of patients with HRD test. Values are reported as frequency (n [%]) or as mean (range).

Characteristic	Patient number (percent, %)
Age, years	
<55	33 (49.3)
≥55	34 (50.7)
Primary tumor site	
Ovary	65 (97.0)
Fallopian tube	2 (3.0)
FIGO stage	
I	1 (1.5)
II	4 (6.0)
III	49 (73.1)
IV	13 (19.4)
Histological type	
High-grade serous	58 (86.6)
Endometrioid	5 (7.5)
Low-grade serous	1 (1.5)
Clear cell carcinoma	1 (1.5)
Carcinosarcoma	1 (1.5)
Well-differentiated papillary mesothelioma	1 (1.5)
Residual disease after primary surgery	
R0	29 (43.3)
R1	32 (47.8)
R2	6 (9.0)
ECOG PS	
0	37 (55.2)
1	30 (44.8)
Neoadjuvant chemotherapy	
Yes	23 (34.3)
No	44 (65.7)
Treatment lines	
≤2	27 (40.3)
≥3	40 (59.7)
Treatment categories	
First-line maintenance therapy	8 (11.9)
Second-line maintenance therapy	6 (9.0)
Multi-line monotherapy	21 (31.3)
Exploratory therapy	
First-line monotherapy	2 (3.0)
First-line maintenance therapy	5 (7.5)
Second-line monotherapy	4 (6.0)
Second-line maintenance therapy	2 (3.0)
Multi-line monotherapy	19 (28.4)
Secondary cytoreductive surgery	
Yes	18 (26.9)
No	49 (73.1)
Family history	
Yes	24 (35.8)
No	43 (64.2)
BRCA status	
BRCAm	24 (35.8)
BRCAw	43 (64.2)
HRD status	
positive	46 (68.7)
negative	21 (31.3)
Platinum sensitivity	
Yes	17 (25.4)
No	32 (47.8)
Unknown	18 (26.9)
PARP inhibitor	
Olaparib	47 (70.1)
Niraparib	20 (29.9)

HRD, homologous recombination deficiency; FIGO, International Federation of Gynecology and Obstetrics; ECOG PS, Eastern Cooperative Oncology Group performance status; R0, no macroscopic disease; R1, 1 cm or less; R2, more than 1 cm; PDS, primary debulking surgery; IDS, interval debulking surgery; NACT, Neoadjuvant chemotherapy.

### HRD Status Association With BRCA Mutation, HRR Mutation in Ovarian Cancer Patients

Approximately 35.8% (24/67) patients were BRCA mutation positive, and 41.8% (28/67) were HRR mutation positive. Approximately 68.7% (46/67) of the patients were HRD positive and the rest of the 31.3% (21/67) were HRD negative. Among the HRD positive patients, 52.2% (24/46) were BRCA mutation positive and 47.8% (22/46) were BRCA mutation negative. Both the HRD-positive and BRCA-positive populations are higher than the western population reported in QUADRA, PRIMA, and PAOLA-1 trials where the HRD-positive patients represent around 50% of western ovarian cancer patients (6, 10, 12). Meanwhile, recently, the ASCO2021 reported the L-MOCA: an open-label study of olaparib maintenance monotherapy in platinum-sensitive relapsed ovarian cancer in Chinese and Malaysian patients, where 47.3% (106/224) of the patients were BRCA mutation positive (20).

Further, we analyzed whether HRD score above cut-off (HRD score ≥42) patients were enriched in some patients with specific BRCA mutation or HRR pathway mutation. The results showed that patients with high HRD scores tend to be enriched in BRCA mutation and HRR mutation. However, the P-value is not significant due to the small sample size. The detailed results are shown in **Supplementary Table 2**.

### HRD Status and Clinical Outcomes of Ovarian Cancer Patients Treated With PARPi

**Figure 2:**
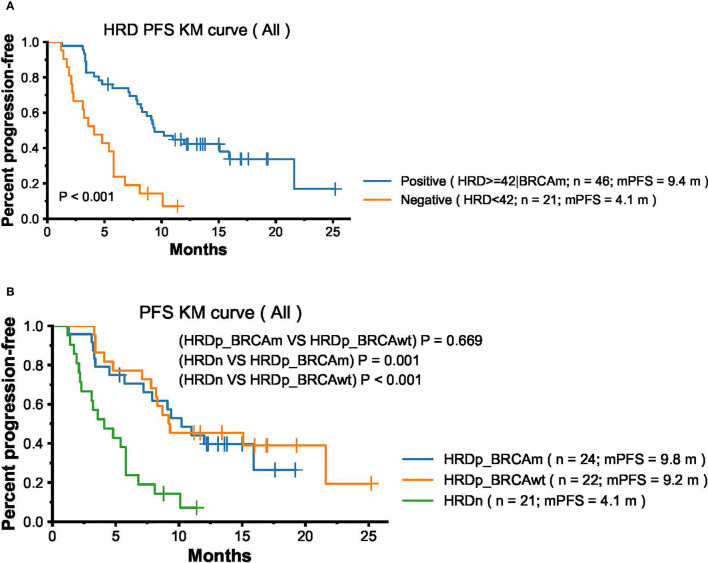
Association Between HRD status and PFS in Ovarian Cancer Patients treated with PARPi olaparib and niraparib. **(A)** Progression-free survival (PFS) by HRD status positive (N = 46) and negative (N = 21). **(B)** Progression-free survival (PFS) by three group: BRCA mutation HRD positive group (N = 24), BRCA wildtype HRD positive (N = 22), and HRD negative (N = 21).

To unravel whether HRD status could identify patients benefiting from PARPi treatment, we compared the PFS difference between HRD positive patients (HRD score ≥42 or BRCA mutation positive, n = 46) and HRD negative patients (HRD score <42, n = 21). The PFS among HRD positive patients was significantly longer than those HRD negative patients (medium PFS 9.4 months *vs* 4.1 months, HR: 0.52, 95% CI [0.38–0.71], p <0.001) (**Figure 2A**). We further defined the HRD-positive group as having two subgroups: the BRCA mutation group (N = 24) and BRCA wildtype HRD positive (N = 22). We can see that both the BRCA mutation group and BRCA wildtype HRD-positive group showed significant higher PFS than the HRD-negative group (BRCAm: medium PFS 9.8 months vs 4.1 months, HR: 0.29, 95% CI[0.14–0.61], p = 0.001 and BRCAw HRD positive: medium PFS 9.2 months vs 4.1 months, HR: 0.52, 95% CI[0.36–0.76], p <0.001, respectively). The PFS difference between the BRCA mutation group and the BRCA wildtype HRD-positive group is not significant (PFS 9.8 months vs 9.2 months, HR: 0.85, 95% CI [0.40–1.80], p = 0.67) (**Figure 2B**).

In the univariable Cox proportional hazards regression model, in addition to the HRD status, Eastern Cooperative Oncology Group (ECOG) were also associated with PFS (ECOG: HR, 2.49; 95% CI [1.39–4.44];* *p = 0.002). We further explored the association between HRD status and PFS in different ECOG statuses. The HRs and P-values still remained significant in better ECOG performance group (medium PFS 12.0 months *vs* 4.9 months; HR: 0.36; 95% CI [0.22–0.59]; p <0 .001) while the PFS in worse ECOG performance group were generally poor regardless of HRD status (medium PFS 4.5 months *vs* 3.2 months; HR: 0.79; 95% CI [0.52–1.18]; p = 0.23) (**Supplementary Figures 1A–C**).

Treatment lines, BRCA status, secondary cytoreductive surgery and R0 resection or not also tend to be associated with PFS (treatment lines: HR, 1.58; 95% CI [0.87–2.87]; *P*  = 0 .13; BRCA status: HR, 0.66; 95% CI [0.36–1.23]; *P*  = 0.19; Secondary cytoreductive surgery: HR, 1.59; 95% CI [0.85–2.98]; *P* = 0 .15; R0 resection or not: HR, 1.54; 95% CI [0.86–2.77]; *P* = 0 .15). Baseline variables that achieved a level of significance of* *P < 0.2 in the univariable analysis were entered into multivariable models. In the multivariate Cox proportional hazards regression model that included the above six factors, the association between HRD status and ECOG status remained significant (HRD status: HR: 0.67; 95% CI, [0.49–0.92]; p = 0.01; ECOG: HR: 2.20; 95% CI, [1.14–4.23]; p = 0.02) (**Table 2** and **Supplementary Figure 2**).

**Table 2:** Univariable and Multivariable Analysis of Progression-Free Survival for the total 67 patient cohort (N = 67).

Parameter	Univariable Analysis		Multivariable Analysis	
	HR (95% CI)	*P*-Value	HR (95% CI)	*P-*Value
HRD Status	0.60 (0.45–0.82)	<0.001	0.67 (0.49–0.92)	0.01
HRR mutation status	0.84 (0.47–1.50)	0.55	NA	NA
BRCA mutation status	0.66 (0.36–1.23)	0.19	0.74 (0.39–1.42)	0.37
ECOG	2.49 (1.39–4.44)	0.002	2.20 (1.14–4.23)	0.02
NACT	1.45 (0.81–2.61)	0.21	NA	NA
Treatment Lines	1.58 (0.87–2.87)	0.13	1.16 (0.61–2.20)	0.64
Family History	0.71 (0.39–1.32)	0.28	NA	NA
Secondary cytoreductive surgery	1.59 (0.85–2.98)	0.15	1.80 (0.91–3.53)	0.09
R0 resection or not	1.54 (0.86–2.77)	0.15	1.75 (0.96–3.26)	0.07
Stage	0.72 (0.42–1.23)	0.23	NA	NA

ECOG, Eastern Cooperative Oncology Group; ECOG performance status ≥2 vs 1 or 0; NACT, New Adjuvant Chemo Therapy yes or no; HR, hazard ratio; Treatment lines, lines ≤2 as 0, ≥3 lines as 1; NA, not applicable. Baseline variables that achieved a level of significance of P <0 .2 in the univariable analysis were entered into multivariable models.

Since HRD score was the sum of LOH, TAI or LST, we further analyzed that LOH, TAI or LST independently correlated with PFS status. Each median score of LOH, TAI or LST was used as the threshold, with the PFS among high LST score group was significantly longer than those low LST score group (medium PFS 10.1 months *vs* 5.6 months, HR: 0.95, 95% CI [0.93–0.98], logRank p = 0.02) (**Supplementary Figure 3A** and **Supplementary Table 3**). There was a trend that PFS among high TAI or LOH score group was longer than those low TAI or LOH score group, although the p-value is not significant (**Supplementary Figures 3B, C**). Univariable Cox proportional hazards regression model showed that LOH, TAI, and LST were all associated with PFS (LOH: HR, 0.95; 95% CI [0.90–0.99]; p  = 0 .026; TAI: HR, 0.95; 95% CI [0.91–0.98]; p = 0 .005; LST: HR, 0.95; 95% CI [0.93–0.98]; p <0 .001) (**Supplementary Table 3**). However, after multivariate regression correction, only LST status association with PFS remained significant (HR: 0.95; 95% CI [0.90–0.996]; p =  0.03) (**Supplementary Table 3**). From this dataset, we found that LOH, TAI and LST are not independent variables correlated with PFS and LST was the most significant.

### Platinum Sensitivity Evaluable Subgroup Analysis

In the subgroup PARPi treated as first-line maintenance therapy and some exploratory therapy, platinum sensitivity status was not evaluable. However, platinum sensitivity status was recognized as a clinical marker to predict PARPi response in the second-line maintenance therapy. Thus we chose the platinum sensitivity evaluable subgroup (N = 49) to further analyze the HRD status, platinum sensitivity and other clinical factors associated with PFS of PARPi treatment. In the univariable Cox proportional hazards regression model, the HRD status, ECOG and platinum sensitivity were associated with PFS (HRD: HR, 0.70; 95% CI [0.51–0.96]; p = 0.03; ECOG: HR, 1.87; 95% CI [0.99–3.51]; p = 0.05; platinum sensitivity: HR, 0.47; 95% CI [0.24–0.94]; p = 0 .03). R0 resection or not tended to be associated with PFS (HR: 1.70; 95% CI [0.89–3.24]; p = 0 .11). In the multivariate Cox proportional hazards regression model that included the above four factors, the association between HRD status, and platinum sensitivity status remained significant (HRD status: HR, 0.71; 95% CI, [0.51–0.98]; p = 0.04; platinum sensitivity: HR, 0.49; 95% CI, [0.24–1.0]; p = 0.05) (**Table 3** and **Supplementary Figure 4**).

**Table 3:** Univariable and Multivariable Analysis of Progression-Free Survival for the platinum sensitivity status evaluable subgroup (N = 49).

Parameter	Univariable Analysis		Multivariable Analysis	
	HR (95% CI)	*P* Value	HR (95% CI)	*P* Value
HRD Status	0.70 (0.51–0.96)	0.03	0.71 (0.51–0.98)	0.04
HRR mutation status	0.75 (0.39–1.43)	0.38	NA	NA
BRCA mutation status	0.68 (0.35–1.33)	0.26	NA	NA
ECOG	1.87 (0.99–3.51)	0.05	1.55 (0.81–2.97)	0.18
NACT	1.39 (0.73–2.62)	0.31	NA	NA
Treatment Lines	1.30 (0.70–2.44)	0.41	NA	NA
Family History	0.81 (0.41–1.60)	0.55	NA	NA
Secondary cytoreductive surgery	1.31 (0.68–2.51)	0.42	NA	NA
R0 resection or not	1.70 (0.89–3.24)	0.11	1.69 (0.87–3.27)	0.12
Stage	0.87 (0.40–1.91)	0.73	NA	NA
Platinum sensitivity	0.47 (0.24–0.94)	0.03	0.49 (0.24–1.0)	0.05

ECOG, Eastern Cooperative Oncology Group; ECOG performance status ≥2 vs 1 or 0; NACT, New Adjuvant Chemo Therapy yes or no; HR, hazard ratio; Treatment lines, lines ≤2 as 0, ≥3 lines as 1; NA, not applicable. Baseline variables that achieved a level of significance of P <0 .2 in the univariable analysis were entered into multivariable models.

## Discussion

Epithelial ovarian cancer (EOC) has the highest mortality and morbidity in ovarian cancer (1). Germline pathogenic variants in EOC susceptibility genes, namely, those involved in homologous recombination and mismatch repair pathways are present in approximately 22 to 25% of EOC (21). High-grade serous ovarian cancer (HGSOC) is the most common type of EOC, accounting for 75% of all EOC, 15–20% of which western patients have germline BRCA1 or BRCA2 mutations. The BRCA mutation rate observed in our cohort was 35.8%, which was near the higher boundary of the previously reported range of 5 to 35% (14, 22). Similar to other studies, we also observed BRCA1 mutations occurring more frequently than BRCA2 mutations in Chinese ovarian cancer patients (23, 24). HRD is the first phenotypically defined predictive marker for therapy with PARP inhibitors in EOC (25). Genomic analyses show that homologous recombination is defective in nearly half of HGSOC (13, 14). To our knowledge, our study is the first real world study of NGS-based HRD in Chinese ovarian cancer and fallopian tube cancer patients. The proportion of HGSOC patients with HRD observed in our enrolled cohort was 68.7%, which was higher than the HRD positive proportion reported in western countries (50–60%) (14). Homologous recombination repair (HRR), namely, BRCA1, BRCA2, ATM, BARD1, BRIP1, CHEK1, CHEK2, RAD51C, and RAD51D, is an important pathway for normal cells to repair DNA DSB. HRR related mutations can induce HRD. Our results demonstrated that patients with high HRD scores tended to enrich in BRCA mutation and HRR mutation.

In our study, the HRD score was calculated by the Myriad myChoice^®^CDx test method, which was the sum of three HRD events: LOH, TAI, and LST. An HRD score cut-off of 42 represented the 95th percentile of BRCAness samples. BRCAness was defined as the set of known BRCA deficiency including the following three events: (i) one deleterious mutation in BRCA1 or BRCA2, with LOH in the wild-type copy, (ii) two deleterious mutations in the same gene, or (iii) promoter methylation of BRCA1 with LOH in the wild-type copy (26). From AmoyDx assay validation data, HRD score cut-off of 42 represented the 93th percentile of the set of BRCAness in Chinese ovarian cancer patients (N = 200) (Data was not shown in this paper). Previous studies demonstrated that LOH, TAI, and LST all proved to be useful markers in predicting response to a variety of therapeutic strategies exploiting defective DNA repair (19). Univariable Cox proportional hazards regression model showed that LOH, TAI, and LST were all associated with PFS and LST status association with PFS remaining the most significant after multiple regression correction.

The approval of PARPi is an important milestone in the treatment of ovarian cancer patients. Many clinical studies have confirmed that ovarian cancer patients with BRCAmt and HRD positive can benefit from PARPi. Olaparib as maintenance treatment significantly increased PFS for patients with BRCAmt in SOLO1 and SOLO2 study (5, 27). Both NOVA and PRIMA studies found that patients with HRD positive could get more profit from niraparib as maintenance treatment (4, 6). The QUADRA study demonstrated that niraparib works among women with heavily pretreated ovarian cancer, especially in patients with HRD-positive platinum-sensitive disease, which included not only patients with BRCA mutation but also the population with BRCA wild-type (10). In the Chinese population, the NORA study found that niraparib maintenance treatment reduced the risk of disease progression or death by 68% and prolonged PFS in patients with platinum-sensitive recurrent ovarian cancer (28). However, a limited number of studies have investigated the relationship between HRD status and efficacy of PAPRi in the Chinese population. Our study performed the largest retrospective study to detect HRD status from Chinese ovarian cancer patients and correlate HRD status and clinical characteristics with PARPi therapeutic outcomes. However, PARP inhibitors have been available in China not for a long time, most of the patients we enrolled were taking it as multi-line monotherapy, which led to shorter PFS of our cohort.

Very consistent results were observed in both our cohort and the western population; whereby Chinese ovarian cancer patients with positive HRD had significantly better PFS after PARPi treatment. The single variant Cox proportional hazards regression analysis showed that the efficacy of PARPi was closely related to HRD status, ECOG score, treatment lines, BRCA mutation status, secondary tumor cell reduction and R0 resection or not. The multivariable analysis highlighted that HRD and ECOG were independent factors affecting the prognosis. Further analysis showed that the PFS in better ECOG performance group was significantly higher in HRD positive population while the PFS in worse ECOG performance group were generally poor regardless of HRD status. In the subgroup with the platinum sensitivity evaluable, HRD and platinum sensitivity were consistently significant to predict PFS no matter in univariable or multivariant regression analysis. However, BRCAmt was only significant to predict PFS in the univariable analysis. It was not statistically significant to the association between BRCAmt and PFS in the multivariate analysis, which may be due to a small cohort and more patients with multi-line monotherapy that underpowered statistical procedure.

In conclusion, our study highlights several important considerations. We firstly elucidated the HRD status of ovarian cancer patients from China in the real world. Chinese ovarian cancer patients with HRD positive also had a better response to PARPi therapy and our cohort also found other clinical characteristics that could affect the efficacy of PARPi. Studies with larger cohorts are needed to validate these observations to expand its therapeutic markers.

## Data Availability Statement

The data and materials used in this manuscript is available from the corresponding author on reasonable requests.

## Ethics Statement

The studies involving human participants were reviewed and approved by The Affiliated Cancer Hospital of Nanjing Medical University. The patients/participants provided their written informed consent to participate in this study.

## Author Contributions

JN and WG participated in the design of the present study and drafted the manuscript. QZ carried out the case recruitment of the present study. XZC and XX participated in the case recruitment of the present study. RZ and HG carried out statistical analysis. WG and CC participated in the statistical analysis. XXC designed of the study, performed the statistical analysis and revised the manuscript. All authors contributed to the article and approved the submitted version

## Funding

This study was supported by grants from the National Natural Science Foundation of China 353 (No. 81472441, 81501205), Natural Science Foundation of Youth Fund Projects of Jiangsu Province (SBK2021040731), Jiangsu Provincial Scientific research and Health Project for Women and Children (No. F202004), Institute level project of Jiangsu Cancer Hospital (No. 355 ZM201804) and Beijing Kanghua Foundation for the Development of Traditional Chinese and Western Medicine -Le Fund (KH-2020-LJJ-021, KH-2021-LLZX-058).

## Conflict of Interest

The authors declare that the research was conducted in the absence of any commercial or financial relationships that could be construed as a potential conflict of interest.

## Publisher’s Note

All claims expressed in this article are solely those of the authors and do not necessarily represent those of their affiliated organizations, or those of the publisher, the editors and the reviewers. Any product that may be evaluated in this article, or claim that may be made by its manufacturer, is not guaranteed or endorsed by the publisher.

## Supplementary Material

Supplementary Figure 1 Comparison of Progression-Free Survival Between **(A)** ECOG score. Comparison of Progression-Free Survival Between HRD positive and negative group in **(B)** ECOG score=0 subgroup; **(C)** ECOG score=1 subgroup.

Supplementary Figure 2 Figure presentation of the multivariate Cox proportional hazards regression model log(HR) and 95% Confidence Interval from the six factors associated with PFS (N=67).

Supplementary Figure 3 Comparison of Progression-Free Survival Between Patients in the **(A)** LST high and LST low group; **(B)** TAI high and TAI low group; **(C)** LOH high and LOH low group. Each median score of LOH, TAI or LST was used as the threshold.

Supplementary Figure 4 Figure presentation of the multivariate Cox proportional hazards regression model log(HR) and 95% Confidence Interval from the four factors associated with PFS in the platinum sensitivity evaluable subgroup (N=49).

## Abbreviations

HRD, homologous recombination deficiency; PARPi, Poly (ADP-ribose) polymerase inhibitors; PFI, platinum free interval; LOH, loss of heterozygosity; TAI, telomeric allelic imbalance; LST, large-scale state transitions; NGS, next generation sequencing; PFS, progression free survival; SCS, secondary cytoreductive surgery; ECOG PS, Eastern Cooperative Oncology Group performance status; FIGO, International Federation of Gynecology and Obstetrics; R0, no macroscopic disease after surgery; R1, 1 cm or less macroscopic disease after surgery; R2, more than 1 cm macroscopic disease after surgery; PDS, primary debulking surgery; IDS, interval debulking surgery; NACT, Neoadjuvant chemotherapy; VUS, variants of unknown significance.
