# Supplementary figures and images for: Homologous Recombination Deficiency Associated With Response to Poly (ADP-ribose) Polymerase Inhibitors in Ovarian Cancer Patients: The First Real-World Evidence From China
Source: Front Oncol. 2022 Jan 6;11:746571. doi: 10.3389/fonc.2021.746571 (PMC8779205; doi:10.3389/fonc.2021.746571)

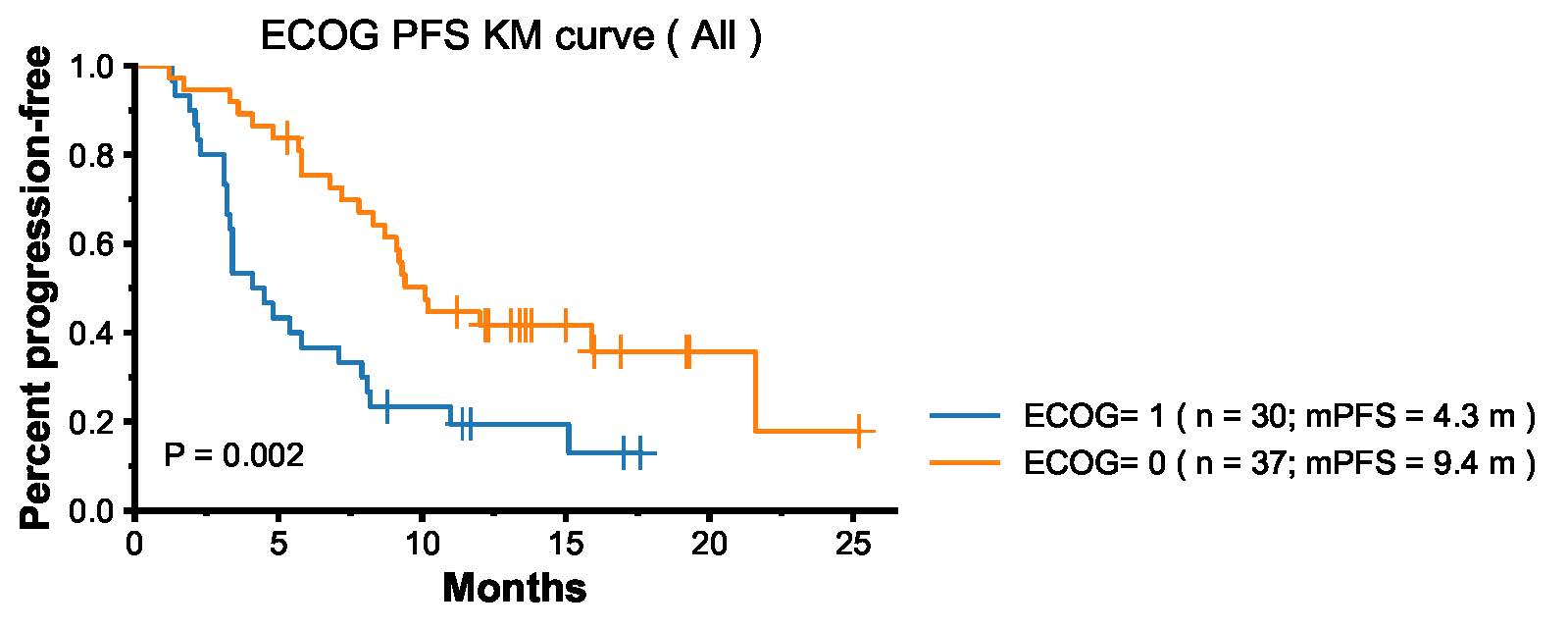

Supplement: Supplementary Figure 1 — Comparison of Progression-Free Survival Between (A) ECOG score. Comparison of Progression-Free Survival Between HRD positive and negative group in (B) ECOG score=0 subgroup; (C) ECOG score=1 subgroup. [file DataSheet_1.zip › Supplementary Images/SFig1A_ECOG_All.jpg]

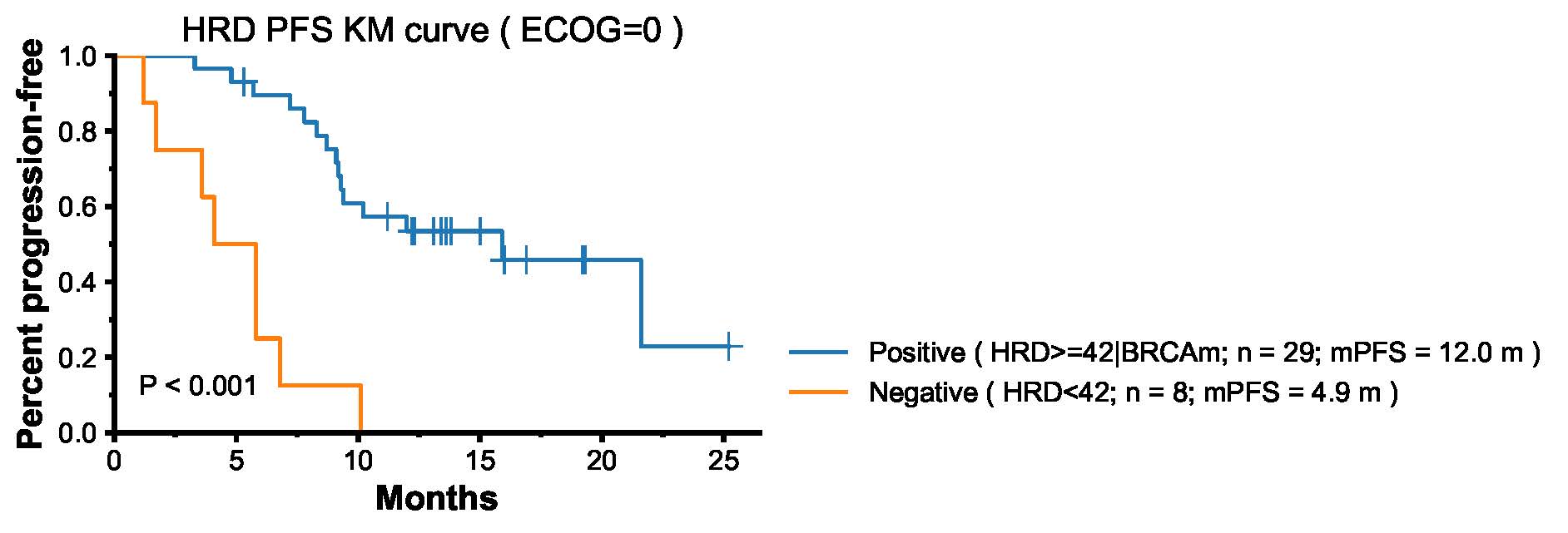

Supplement: Supplementary Figure 1 — Comparison of Progression-Free Survival Between (A) ECOG score. Comparison of Progression-Free Survival Between HRD positive and negative group in (B) ECOG score=0 subgroup; (C) ECOG score=1 subgroup. [file DataSheet_1.zip › Supplementary Images/SFig1B_HRD_Blabel2_ECOG=0.jpg]

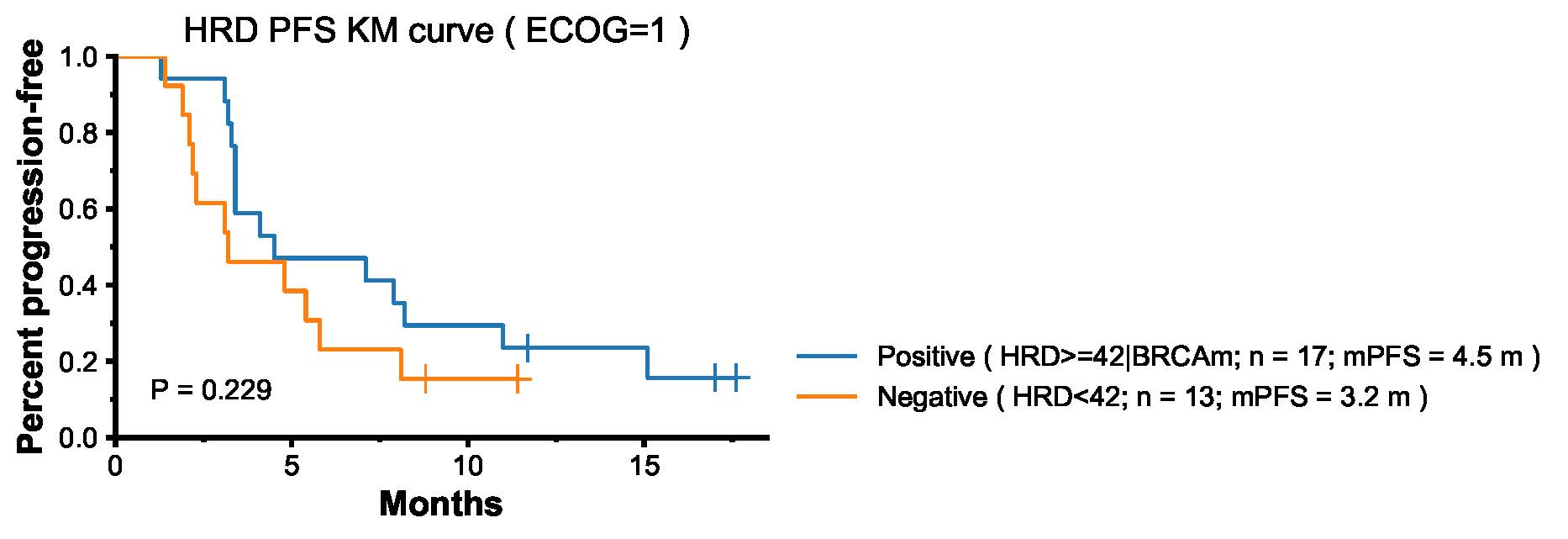

Supplement: Supplementary Figure 1 — Comparison of Progression-Free Survival Between (A) ECOG score. Comparison of Progression-Free Survival Between HRD positive and negative group in (B) ECOG score=0 subgroup; (C) ECOG score=1 subgroup. [file DataSheet_1.zip › Supplementary Images/SFig1C_HRD_Blabel2_ECOG=1.jpg]

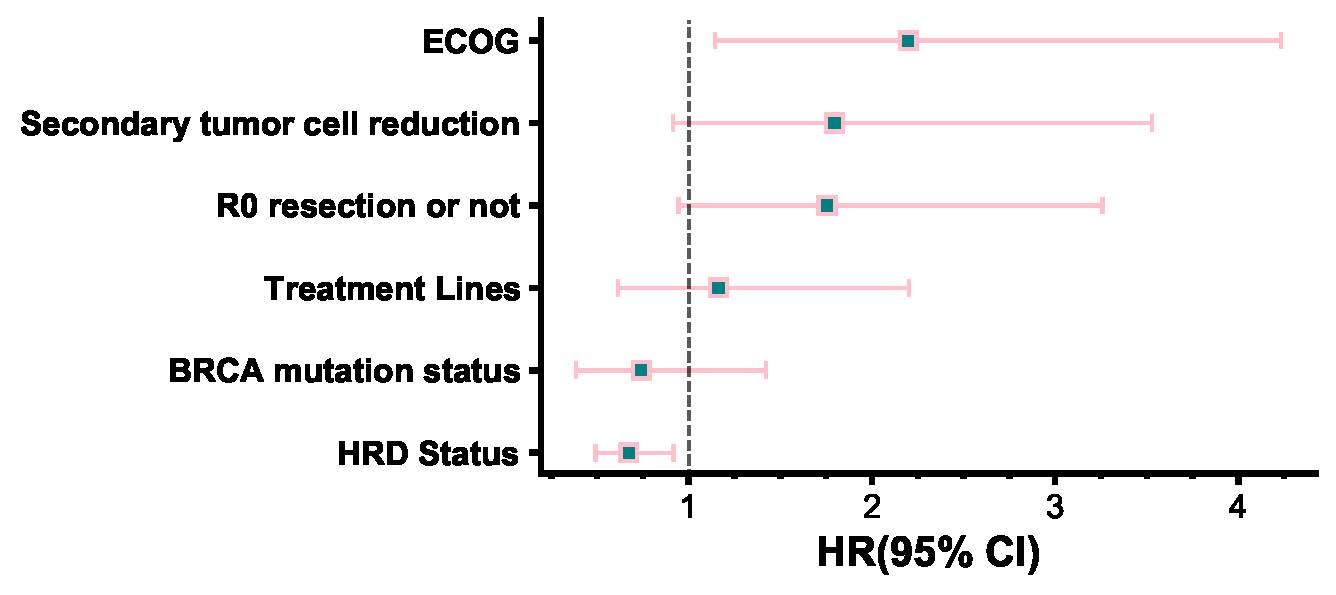

Supplement: Supplementary Figure 1 — Comparison of Progression-Free Survival Between (A) ECOG score. Comparison of Progression-Free Survival Between HRD positive and negative group in (B) ECOG score=0 subgroup; (C) ECOG score=1 subgroup. [file DataSheet_1.zip › Supplementary Images/SFig2_HR(CI95)_67.jpg]

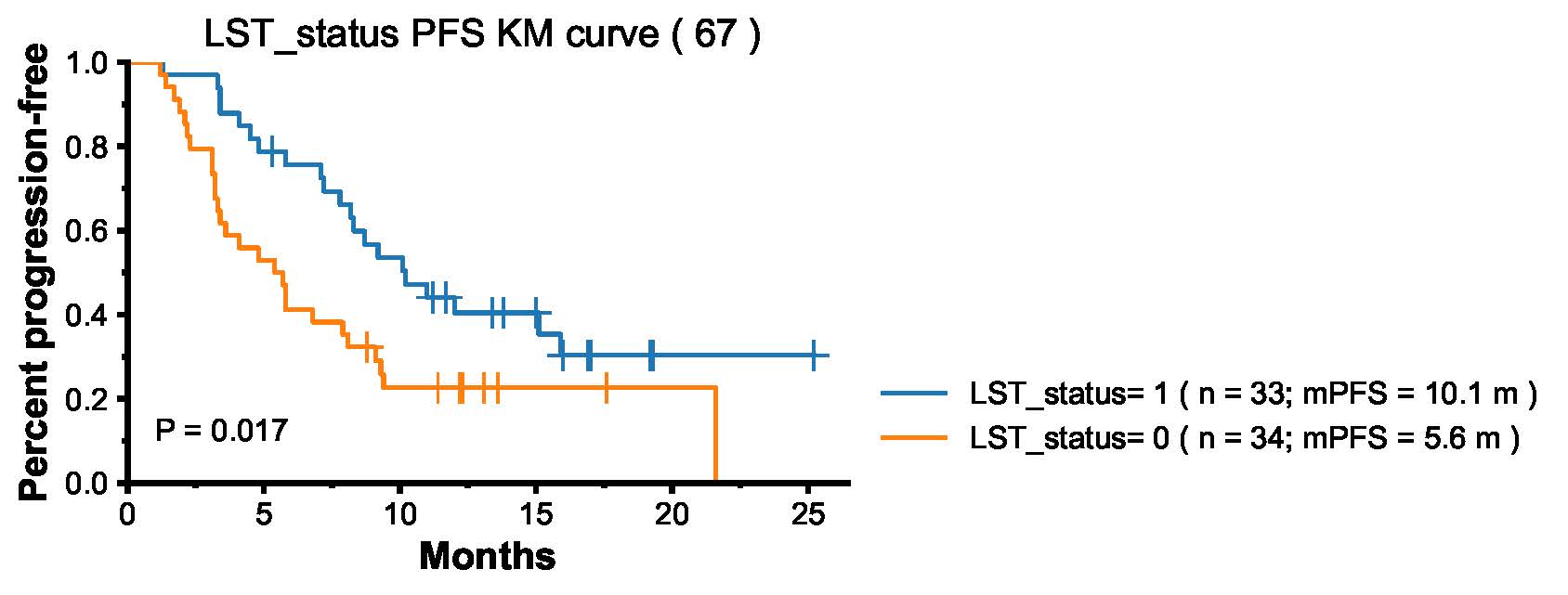

Supplement: Supplementary Figure 1 — Comparison of Progression-Free Survival Between (A) ECOG score. Comparison of Progression-Free Survival Between HRD positive and negative group in (B) ECOG score=0 subgroup; (C) ECOG score=1 subgroup. [file DataSheet_1.zip › Supplementary Images/SFig3A_LST_status_67.jpg]

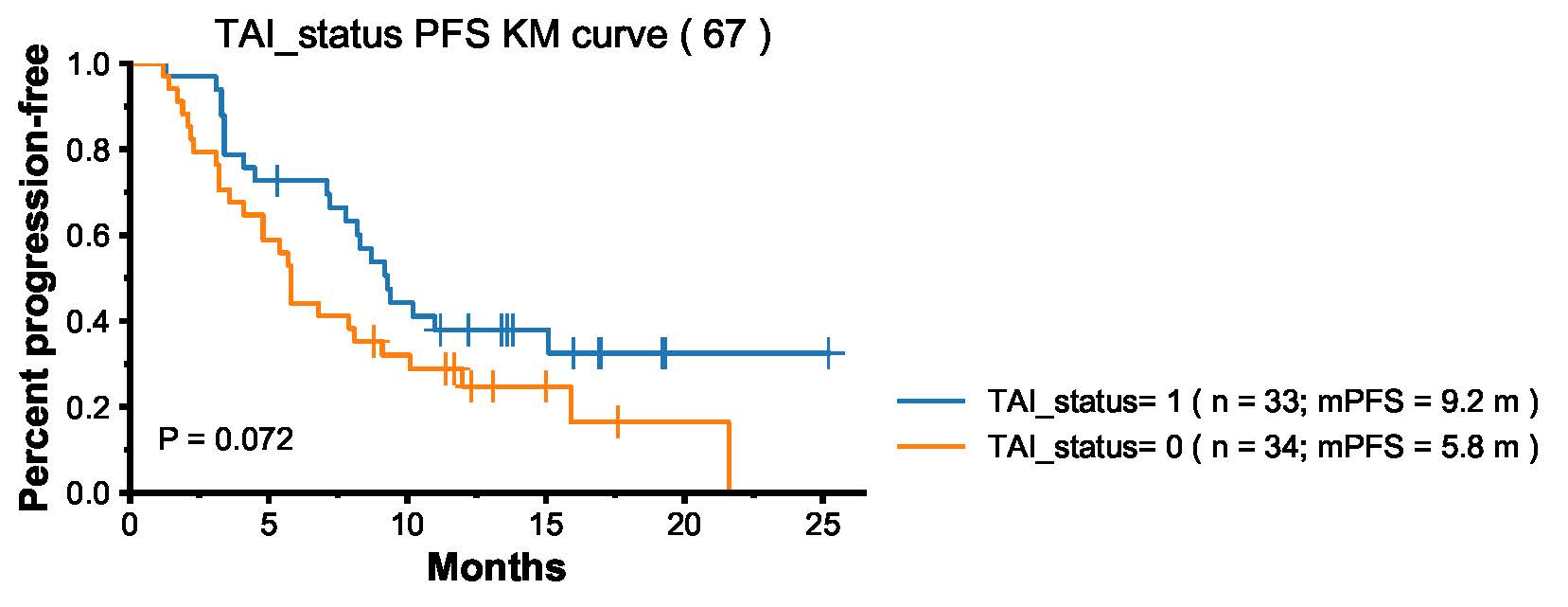

Supplement: Supplementary Figure 1 — Comparison of Progression-Free Survival Between (A) ECOG score. Comparison of Progression-Free Survival Between HRD positive and negative group in (B) ECOG score=0 subgroup; (C) ECOG score=1 subgroup. [file DataSheet_1.zip › Supplementary Images/SFig3B_TAI_status_67.jpg]

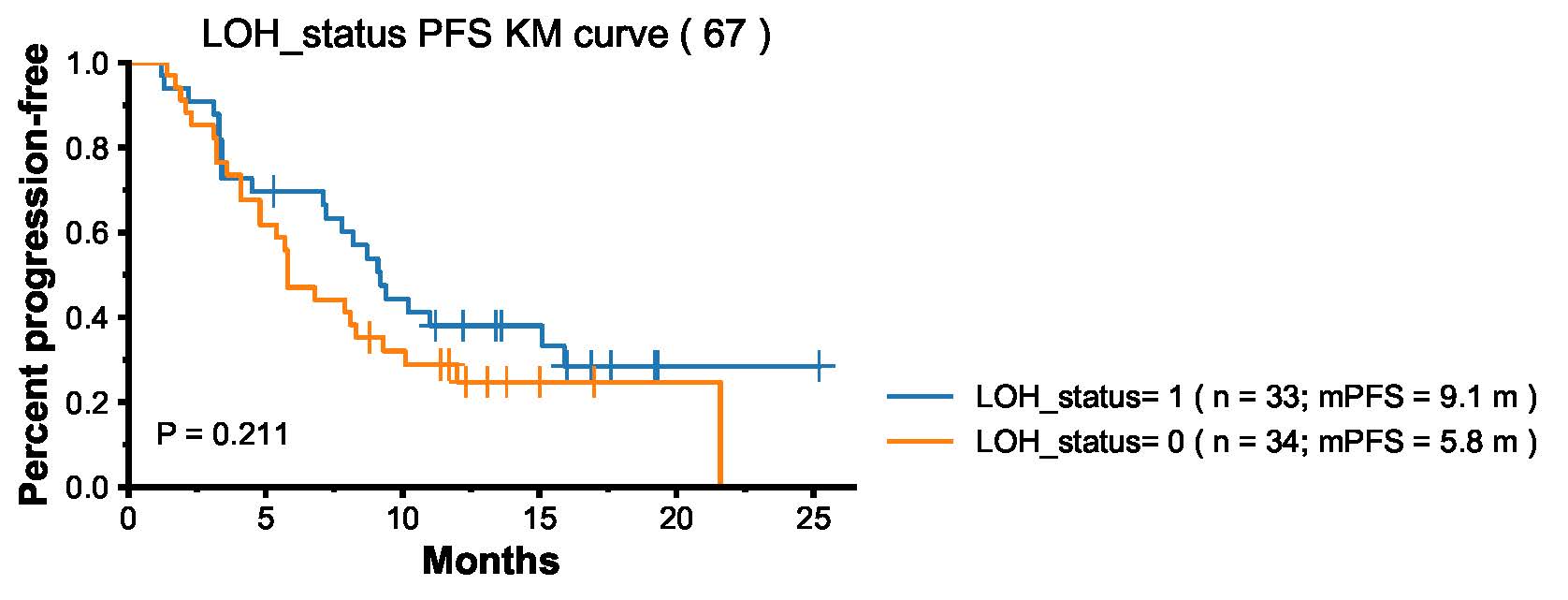

Supplement: Supplementary Figure 1 — Comparison of Progression-Free Survival Between (A) ECOG score. Comparison of Progression-Free Survival Between HRD positive and negative group in (B) ECOG score=0 subgroup; (C) ECOG score=1 subgroup. [file DataSheet_1.zip › Supplementary Images/SFig3C_LOH_status_67.jpg]

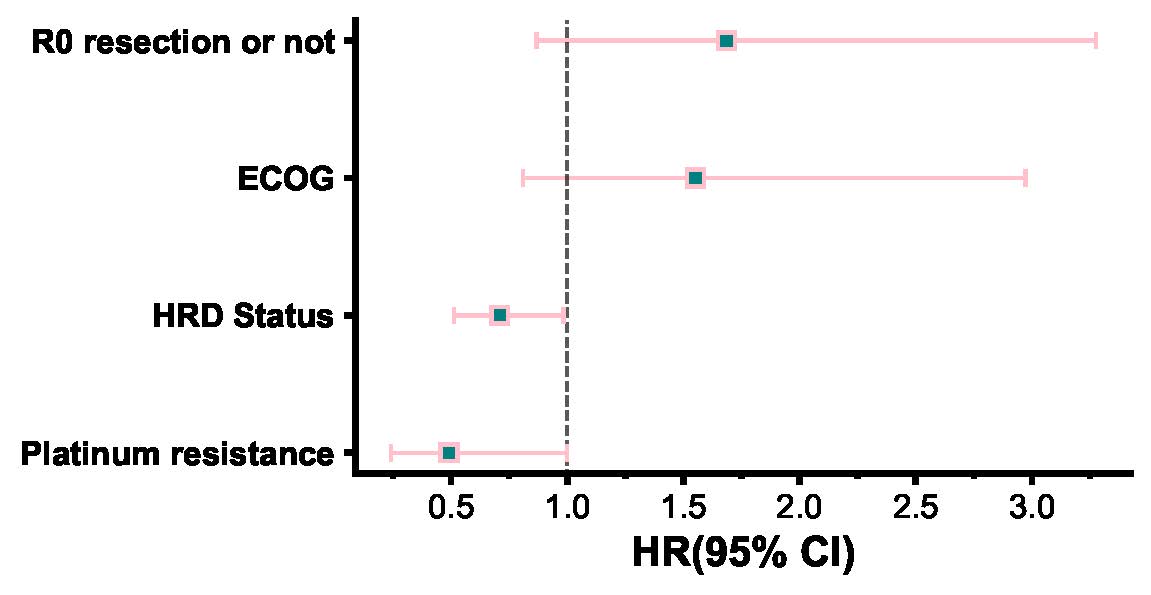

Supplement: Supplementary Figure 1 — Comparison of Progression-Free Survival Between (A) ECOG score. Comparison of Progression-Free Survival Between HRD positive and negative group in (B) ECOG score=0 subgroup; (C) ECOG score=1 subgroup. [file DataSheet_1.zip › Supplementary Images/SFig4_HR(CI95)_49.jpg]
