# Supplementary material for: Homologous Recombination Deficiency Associated With Response to Poly (ADP-ribose) Polymerase Inhibitors in Ovarian Cancer Patients: The First Real-World Evidence From China
Source: Front Oncol. 2022 Jan 6;11:746571. doi: 10.3389/fonc.2021.746571 (PMC8779205; doi:10.3389/fonc.2021.746571)
Supplement: Supplementary file 2 [file Table_1.docx]

**Supplementary Table 1: 54 gene list**

| *Gene* | transcript |
| --- | --- |
| *ATM* | NM_000051.3 |
| *ATR* | NM_001184.3 |
| *ATRX* | NM_000489.4 |
| *BAP1* | NM_004656.4 |
| *BARD1* | NM_000465.3 |
| *BLM* | NM_000057.3 |
| *BRCA1* | NM_007294.3 |
| *BRCA2* | NM_000059.3 |
| *BRIP1* | NM_032043.2 |
| *CDH1* | NM_004360.3 |
| *CDK12* | NM_016507.3 |
| *CHEK1* | NM_001274.5 |
| *CHEK2* | NM_007194.3 |
| *EMSY* | NM_001300942.1 |
| *EPCAM* | NM_002354.2 |
| *FAM175A* | NM_139076.2 |
| *FANCA* | NM_000135.2 |
| *FANCC* | NM_000136.2 |
| *FANCD2* | NM_033084.3 |
| *FANCE* | NM_021922.2 |
| *FANCF* | NM_022725.3 |
| *FANCG* | NM_004629.1 |
| *FANCI* | NM_018193.2 |
| *FANCL* | NM_018062.3 |
| *FANCM* | NM_020937.2 |
| *HDAC2* | NM_001527.4 |
| *KMT2D* | NM_001197104.1 |
| *MDC1* | NM_014641.2 |
| *MLH1* | NM_000249.3 |
| *MRE11* | NM_005591.3 |
| *MSH2* | NM_000251.2 |
| *MSH6* | NM_000179.2 |
| *MUTYH* | NM_001128425.1 |
| *NBN* | NM_002485.4 |
| *NF1* | NM_001042492.2 |
| *PALB2* | NM_024675.3 |
| *PARP1* | NM_001618.3 |
| *PMS1* | NM_000534.4 |
| *PMS2* | NM_000535.5 |
| *PPP2R2A* | NM_002717.3 |
| *PTEN* | NM_000314.4 |
| *RAD50* | NM_005732.3 |
| *RAD51* | NM_002875.4 |
| *RAD51B* | NM_133509.3 |
| *RAD51C* | NM_058216.2 |
| *RAD51D* | NM_133629.2 |
| *RAD52* | NM_001297419.1 |
| *RAD54L* | NM_001142548.1 |
| *SLX4* | NM_032444.2 |
| *STK11* | NM_000455.4 |
| *TP53* | NM_000546.5 |
| *WRN* | NM_000553.4 |
| *XRCC1* | NM_006297.2 |
| *XRCC2* | NM_005431.2 |

**Supplementary Table 2: BRCA, HRR mutation association with HRD score**

| Patients category (N=67) | HRD score  >=42 | | HRD score  <42 | Odds ratio | *P* value |
| --- | --- | --- | --- | --- | --- |
| BRCA1/BRCA2 mutation | 17 | 6 | | 2.55 (95%CI:0.77-9.46) | 0.1173 |
| BRCA1/BRCA2 wildtype | 23 | 21 | |  |  |
| HRR mutation positive | 20 | 8 | | 2.34 (95%CI:0.76-7.73) | 0.1314 |
| HRR wildtype | 20 | 19 | |  |  |

**Supplementary Table 3: LOH, TAI and LST status association with PFS**

| Parameter | Univariable Analysis | | Multivariable Analysis | |
| --- | --- | --- | --- | --- |
|  | HR (95% CI) | *P* Value | HR (95% CI) | *P* Value |
| LOH | 0.95(0.90-0.99) | 0.0264 | 1.05(0.93-1.19) | 0.3991 |
| TAI | 0.95(0.91-0.98) | 0.0045 | 0.96(0.86-1.06) | 0.4037 |
| LST | 0.95(0.93-0.98) | 0.0005 | 0.95(0.90-0.996) | 0.0328 |
